# Innovative approach for transcriptomic analysis of obligate intracellular pathogen: selective capture of transcribed sequences of *Ehrlichia ruminantium*

**DOI:** 10.1186/1471-2199-10-111

**Published:** 2009-12-24

**Authors:** Loïc Emboulé, France Daigle, Damien F Meyer, Bernard Mari, Valérie Pinarello, Christian Sheikboudou, Virginie Magnone, Roger Frutos, Alain Viari, Pascal Barbry, Dominique Martinez, Thierry Lefrançois, Nathalie Vachiéry

**Affiliations:** 1 UMR 15 CIRAD-INRA «Contrôle des maladies animales exotiques et émergentes», Site de Duclos, Prise d'Eau 97170, Petit Bourg, Guadeloupe; 2 Département de microbiologie et immunologie, Université de Montréal, C.P 6128 succursale Centre-ville, Montréal, QC H3C3J7, Canada; 3 UMR6097, CNRS-Université de Nice Sophia Antipolis, Institut de Pharmacologie Moléculaire et cellulaire, Sophia Antipolis, F06560, France; 4 UMR 15 CIRAD-INRA «Contrôle des maladies animales exotiques et émergentes», TA 30/G Campus international de Baillarguet 34398 Montpellier Cedex 5, France; 5 Inria Rhône-Alpes Projet HELIX, 655 Av. de l'Europe, 38330 Montbonnot-Saint Martin, France

## Abstract

**Background:**

Whole genome transcriptomic analysis is a powerful approach to elucidate the molecular mechanisms controlling the pathogenesis of obligate intracellular bacteria. However, the major hurdle resides in the low quantity of prokaryotic mRNAs extracted from host cells. Our model *Ehrlichia ruminantium (ER*), the causative agent of heartwater, is transmitted by tick *Amblyomma variegatum*. This bacterium affects wild and domestic ruminants and is present in Sub-Saharan Africa and the Caribbean islands. Because of its strictly intracellular location, which constitutes a limitation for its extensive study, the molecular mechanisms involved in its pathogenicity are still poorly understood.

**Results:**

We successfully adapted the SCOTS method (Selective Capture of Transcribed Sequences) on the model Rickettsiales *ER *to capture mRNAs. Southern Blots and RT-PCR revealed an enrichment of *ER*'s cDNAs and a diminution of ribosomal contaminants after three rounds of capture. qRT-PCR and whole-genome *ER *microarrays hybridizations demonstrated that SCOTS method introduced only a limited bias on gene expression. Indeed, we confirmed the differential gene expression between poorly and highly expressed genes before and after SCOTS captures. The comparative gene expression obtained from *ER *microarrays data, on samples before and after SCOTS at 96 hpi was significantly correlated (R^2 ^= 0.7). Moreover, SCOTS method is crucial for microarrays analysis of *ER*, especially for early time points post-infection. There was low detection of transcripts for untreated samples whereas 24% and 70.7% were revealed for SCOTS samples at 24 and 96 hpi respectively.

**Conclusions:**

We conclude that this SCOTS method has a key importance for the transcriptomic analysis of *ER *and can be potentially used for other Rickettsiales. This study constitutes the first step for further gene expression analyses that will lead to a better understanding of both *ER *pathogenicity and the adaptation of obligate intracellular bacteria to their environment.

## Background

Elucidating molecular mechanisms that drive the adaptation of obligate intracellular pathogens to their host is crucial to understand their pathogenesis. To date, molecular studies on obligate intracellular bacteria can only be performed *ex vivo *at one time or *in vitro *in host cells. Thus, RNA extraction from infected cell cultures leads to low quantities of prokaryotic mRNAs with short half-lives and a high amount of contaminant eukaryotic RNAs [1,2]. Moreover, in prokaryotic RNA, ribosomal RNAs (rRNAs) represent more than 80% of total RNA, whereas mRNAs represent only 2% of total RNAs. Therefore, high throughput gene expression analysis of obligate intracellular bacteria depends strongly on the quality of mRNAs samples, deprived from ribosomal RNAs and host RNAs. Up to recently, no methods were available to obtain purified obligate intracellular bacteria mRNAs from infected cells. Various methods can be used to monitor the complete set of RNA molecules produced by a microorganism, including both targeted and random approaches. Among the latter are differential expression of customized amplification libraries (DECAL) [3] and Selective Capture Of Transcribed Sequences (SCOTS) [4], techniques that combine polymerase chain reaction (PCR) and subtractive hybridization in order to identify genes that are expressed differentially. DECAL method is a powerful technique that permits global comparisons of bacterial gene expression under various growth conditions. It allows direct determination of differential gene expression by comparison of relative intensity with which PCR probes hybridize with individual colonies. However, this method has the disadvantage of being time-consuming and more complex to implement because of the construction of the Customized Amplification Library (CAL). Moreover, this technique does not assure to cover all the genome and several genes could be not detected, thus compelling to construct more complete CALs. Selective capture of transcribed sequences (SCOTS) was initially developed by Graham and Clark-Curtiss in 1999 for the non obligatory intracellular pathogen *Mycobacterium tuberculosis *and allowed to enlighten bacterial gene expression from different growth conditions in macrophages cells. It was also later used for *Salmonella enterica *serovar Typhi [5] and then used successfully for further transcriptomic microarray analysis [6]. Recently, SCOTS was employed to identify the *in vivo *expression of several genes of *Actinobacillus pleuropneumoniae *at different developmental stages post infection [7,8] but was never applied on obligate intracellular pathogens.

The Rickettsia *Ehrlichia ruminantium *(*ER*), (previously *Cowdria ruminantium*) is the causative agent of heartwater, which affects both wild and domestic ruminants and is transmitted by ticks of the genus *Amblyomma *[9]. Heartwater represents a serious problem for livestock productivity in endemic areas such as sub-Saharan Africa and the West-indies and it poses a severe threat to livestock in the American continent due to migratory birds and the presence of potential indigenous vector ticks [10,11]. The genotypic heterogeneity of the bacterium leads to difficulties for the generation of an efficient vaccine [12-15]. Little is known about the genetic determinants and molecular mechanisms of *ER *pathogenesis, due to its isolated intracellular location. *ER *was considered to be a good model to evaluate the feasibility of SCOTS method for obligate intracellular bacteria. Indeed, *ER *is a gram negative bacterium that belongs to alpha proteobacteria and is an obligate intracellular pathogen that infects the endothelium of all blood vessels. *ER *has a complex life cycle described as chlamydia-like developmental cycle [16]. In the early stage of the cycle, elementary bodies, which represent the extracellular and infectious forms of the parasite, adhere to host target cells and then are engulfed. They remain within intracytoplasmic vacuoles, where they divided by binary fission to produce intermediate bodies and further reticulated bodies. After 4 to 6 days, the disruption of host cell leads to the release of numerous elementary bodies thus initiating a new infectious cycle. The genomic organization of this microorganism was revealed by the genome sequencing of two strains: Gardel and Welgevonden [17]. Even if comparative genomic studies provided data on the active mechanisms of genome plasticity [18,19], almost 30% of genes had unknown functions and genes involved in virulence, host cell penetration or invasion and intracellular growth processes remain unidentified.

Expression analysis of *ER *genes during life cycle, between attenuated and virulent strains, will allow the identification of the key factors involved in virulence mechanisms and the development of the bacteria. From the sequencing of Gardel and Welgevonden strains, whole genome *ER *microarrays were designed in order to validate sample quality obtained by SCOTS method and to evaluate the potential use of this method for further *ER *whole transcriptomic analysis. *In vitro *model using bovine endothelial cells and virulent Gardel strain allowed us to obtain a sufficient amount of *ER *RNA to perform such a study.

In this article, we report for the first time the successful adaptation of the SCOTS method to an obligate intracellular bacterium, *ER*. We demonstrate the efficient isolation of specific bacterial transcripts from total RNA after 3 rounds of capture, with low amounts of 16S ribosomal RNA contaminant. Moreover, besides the use of PCR amplifications, the differential gene expression was still detected by qRT-PCR and microarrays analysis. SCOTS method seems to be crucial for the analysis of gene expression especially at early stage of *ER *development during the lag phase.

## Methods

### Extraction of *ER *RNA from bovine infected cells

The Gardel strain stock was isolated in 1982 in Guadeloupe from a goat infected experimentally with *Amblyomma variegatum *ticks collected from cows [20]. Gardel strain passage 39 and passage 47 were multiplied successively in bovine aorta endothelial (BAE) cells grown in Glasgow minimal essential medium complemented with fetal calf serum, tryptose-phosphate broth, and antibiotics [21] at 37°C, 5% CO2, with a weekly passage on fresh cells [22]. BAE cells were infected with a calibrated inoculum of Gardel strain (1.1 × 10^7 ^elementary bodies per 1.42 × 10^6 ^cells). Estimation of bacterial viability and quantity were both carried out by flow cytometry and fluorescent microscopy using live/dead BacLight Bacterial Viability Kit (Invitrogen, France) [23]. The infected cells were incubated at 37°C in a 5% CO_2 _atmosphere. The supernatant was renewed only 24 h (6 ml) after infection and the cell monolayer was harvested by trypsinization every 24 h and centrifuged at 1700 × g for 5 min at 4°C. When 80% cell lysis was observed, after 120 hours post infection (hpi), supernatant and cellular debris were harvested and then ultra-centrifuged at 20,000 × g for 15 min at 4°C to collect elementary bodies. The pellets were placed in sterile eppendorfs and homogenized in 2.5 ml of TRIzol reagent (Invitrogen). The cells lysed immediately and released RNA and DNA in the supernatant. The samples were immediately stored at -80°C before RNA extraction.

### Extraction of total RNA with TRIzol

For each time of collection after cell lysis (24 to 120 hpi), total RNA extraction procedure was carried out with TRIzol reagent (Invitrogen) according to the manufacturer's instructions. RNA pellets were dissolved in 100 μl of DEPC water and treated with turboDNAse (Ambion, France) according to manufacturer's protocol in order to remove all contaminant DNA. The removal of bacterial genomic DNA (gDNA) contaminant in RNA samples was verified by PCR targeting *pCS20 *gene using primers AB128 and AB129 which amplified specifically a 281 pb of *ER *as described previously [24] (table 1). The quantification of total RNA was performed by fluorimeter using ribogreen reagent (Invitrogen). The yield obtained after the extraction was between 3 to 25 μg. For each time of collection, total RNA samples were pooled in RNase free water at a final concentration of 0.5 μg/μl.

**Table 1:** Primers used for the detection of specific *ER *genes

Primer name	Primer sequence	Target gene or sequence	Product size (bp)	source (references)
*ffh-F2^a^*	5' GGTAGGTCTTCAAGGTGTTGGTAAA 3'	*Ffh*	121	this work
*ffh-R2*	5' AGTTTGAGCTGCAGGACGATATAA 3'			
				
*recA-F1^a,b^*	5' TTGAAAAAGCGTTTGGTCGTG 3'	*recA*	121	this work
*recA-R1*	5' GGGAAACCACCAATACCCAAT 3'			
				
*rpoD-F1^a,b^*	5' CAGAGGGTTGCAATTTCTTGATT 3'	*rpoD*	121	this work
*rpoD-R1*	5' TCTGACCCACCATGTTGCAT 3'			
				
*16S-F1a^ b^*	5' AGCGCAACCCTCATCCTTAG 3'	*rRNA 16S*	121	this work
*16S-R1*	5' AGCCCACCCTATAAGGGCC 3'			
				
*map1gardF^a,b^*	5' CACTTGAAGGAATGCCAGTTTCTC 3'	*map1*	85	this work
*map1gardR*	5' CTTAGGATTTGTAGCATTGATTACTGACACT 3'			
				
*AB128*	5' ACTAGTAGAAATTGCACAATCTAT 3'	*pCS20*	278	Martinez *et al.*, 2004
*AB129*	5' TGATAACTTGGTGCGGGAAATCCTT 3'			
				
*NKpn1-pdN9*	5' GTGGTACCGCTCTCCGTCCGANNNNNNNNN 3'	*Kpn*I	/	Daigle *et al*., 2001
				
*NKpn1*	5' GTGGTACGGCTCTCCGTCCGA 3'	N*Kpn*I tag	200-400	
				
*ARN16SGarF*	5' AACTTGAGAGTTTGATCCTGGCT 3'	*rRNA 16S*	1503	this work
*ARN16SGarR*	5' AGGAGGTAATCCAGCCGCAGGTT 3'			this work
				
*ARN5SGarR*	5' TCTCCCGTGCCTTAAGACAAA 3'	*rRNA 23S*	2935	this work
*ARN23SGarF*	5' TTGATGGATGCCTTGGCGTTAA 3'	*rRNA 5S*		this work

^a^: pair of primers use for RT-PCR

^b^: pair of primers use for qRT-PCR

### *ER *gDNA production and *ER *ribosomal DNA (rDNA) cloning for SCOTS method

Genomic DNA (> 50 μg) from Gardel strain passage 40 was extracted from elementary bodies, as previously described [18,25]. After high speed centrifugation (20,000 × g during 30 min), the pellet of elementary bodies was resuspended in 350 μl of saline phosphate buffer. 150 μl of DNase (1 μg/ml) was added to remove the contaminant bovine DNA from host cells and the samples were incubated at 37°C for 90 min. The treatment was stopped by adding 25 mM of EDTA. Whole bacterial DNA was obtained using QIAamp extraction kit (Qiagen, France) [26].

In *ER*, the organization of the *rrn *operon coding the rRNAs is not canonical as the gene coding the 16S rRNA is 900 kb distant from the 23S-5S gene cluster [17]. Both rRNA 16S and 23S - 5S cluster sequences were amplified using specific primers derived from *ER *genome: rRNA 16SGarF- rRNA 16SGarR for 16S and rRNA5GarR; rRNA23SGarR for 5S-23S cluster (table 1). The PCR products were cloned into the high copy number vector pGEM-T Easy (Promega, France). Competent *Escherichia coli *SURE2 cells were transformed with plasmid containing the inserts and isolated on LB-ampicillin-XGal (5-bromo-4-chloro-3-indol-β-D-galactopyranoside)-IPTG (isopropyl-β-D-thiogalactopyranoside) selective medium. Positive clones were selected and the presence of the insert was checked using the specific primers previously cited. Transformants were grown in LB medium with appropriate antibiotic (Ampicillin 100 μg/ml) and plasmid extraction was carried out with plasmid purification Maxi kit (Qiagen) according to manufacturer's protocol. Up to 300 μg of plasmid pellets were homogenized in TE buffer pH 7.5. Both gDNA and rDNA were quantified by fluorometer using picogreen reagent (Invitrogen).

### Selective capture of transcribed sequences (SCOTS)

For each time of the kinetic, 5 μg of total RNA from Gardel strain passage 39 was reverse transcribed by random priming with Superscript II (Invitrogen) according to manufacturer's instructions. The reverse transcription was done using *Kpn*I-RNA primers (table 1) containing a defined 5' terminal sequence as a tag and a random nonamer at the 3' end as in conditions previously described by [27] and [5]. Second-strand cDNA was synthesized by using Klenow fragment (Biolabs, France). Then, cDNA was amplified by PCR using the specific primers corresponding to the *Kpn*I tag. This corresponded to cDNA before SCOTS (SCOTS 0×). Reactions were prepared using the following PCR conditions: initial denaturation of 3 min at 94°C followed by 25 cycles at 94°C for 45 s, at 60°C for 45 s, at 72°C for 60 s and a final extension of 10 min at 72°C.

Selective capture of bacterial cDNA was done as previously described by [4] and [28]. *ER *gDNA (0.3 μg) was photobiotinylated and then mixed with the rDNA 16S and 23S+5S (0.5 μg of each plasmid pGEMT) in order to block the rRNAs region sites on the gDNA in TE buffer pH 9. The mixture was then sonicated and precipitated in ethanol 100% (2.5 v/v), NaOac 3 M (0.1 v/v) and 1 μl Glycogen (1 μg/ml). The gDNA-rDNA mixture and 5 μg cDNA were denatured separately 3 min at 99°C in 4 μl of hybridization buffer (10 mM EPPS [N-(2-hydroxyethyl) piperzine-N'-3-propanesulfonic acid]/1 mM EDTA) and pre-hybridized at 50°C for 30 min. The temperature of hybridization was evaluated from the percentage of GC of *ER *genome (27%). The prehybridization step allows the hybridization of *ER *rDNA to the gDNA, as it also allows the normalization of bacterial and eukaryotic cDNA by self-hybridization of highly present cDNA [29,30]. Immediately after adding 1 μl of NaCl 1.5 M, the cDNA and bacterial gDNA pre-blocked with rDNA (gDNA-rDNA) were mixed and hybridized for 18 h at 50°C (hybridization step). Hybrids, representing *ER *cDNA fixed to *ER *gDNA, were captured with streptavidin-coated magnetic beads (Dynal 280) according to the manufacturer's protocol. Selective cDNA was then eluted in 100 μl of NaOH 0.4 N, precipitated and amplified by N*Kpn*I specific PCR as described. The PCR products were then visualized by ethidium bromide staining in 1.2% agarose gels (Seakem) in 1 × TAE buffer (40 mM Tris-HCl, 6% acetic acid, 1 mM EDTA, pH8) and purified with PCR purification kit (Qiagen). The initial amount of total RNA before SCOTS method for each time of infection was between 10 to 20 μg and was divided in 2 to 5 tubes with 0.5 μg/tube. After this first round (SCOTS 1×), the tubes corresponding to one condition were pooled and 10 μl to 20 μl of the pooled sample were precipitated depending on the intensity of purified cDNAs from the previous round. Two additional rounds of capture (SCOTS 2× and 3×) were performed for each sample at each time point of infection (24, 48, 72, 96 and 120 hpi) for further microarrays and qRT-PCR analyzes.

### Microarrays experiments

#### a. *ER *Microarrays design

*ER *microarrays (8 × 15 k) used in this study were developed based on long oligo arrays generated by Agilent technology. These arrays contain 60-mer probes corresponding to 936 coding sequences (CDS) of Gardel strain and 909 sequences of Welgevonden strain, including the specific CDSs for each strain determined by the previous annotation of the two genomes [17]. The probes were designed using the following procedure: first, all 60-mer probes were generated from CDS of Gardel and Welgevonden strains using standard thermodynamical constraints (TM ∈ [78, 83] and GC ∈ [20,36]) and a modified version of the OligoArray program [31]. For non specific CDSs, we chose the same probe for the same pair of orthologs between Gardel and Welgevonden strains. Then, we selected 1 or 2 of the most specific probes per gene by minimizing the number of matches to human and bovine mRNAs, extracted from the Ensembl database [32]. The microarrays contained a total of 1800 probes, with 5 replicates per probe, including 28 bovine genes as negative controls. Experimental data and associated microarray designs have been deposited in the NCBI Gene Expression Omnibus (GEO) http://www.ncbi.nlm.nih.gov/geo/ under platforms GPL9697, GPL 9698 and serie GSE19208. Two different labeling procedures have been performed depending on whether the sample was treated or not following the SCOTS procedure.

#### b. cDNA labeling

Five hundred nanograms of total cDNA (from Gardel strain passage 39 at 24 and 96 hpi) from SCOTS procedure were randomly amplified and fluorescently labeled with the BioPrime array CGH Genomic labeling System kit (Invitrogen), by the incorporation of Cy3-dCTP (Amersham Biosciences) and then purified on a MinElute cleanup column (Qiagen). Before hybridization, quantification of Cy3-dCTP incorporation was performed by absorbance measurement at 550 nm. The yield of cDNA labeling and the specific activity always exceeded to 1.65 μg and 9 pmol of Cy3 per cDNA, respectively, according to the manufacturer's recommendations.

#### c. *In vitro *transcription (IVT)

Total RNA was extracted as described above from Gardel strain samples passage 47, at 24 and 96 hpi. Eukaryotic ribosomal RNA was removed from the mixture by using the RiboMinus Transcriptome isolation kit (Invitrogen), according to the manufacturer's protocol. The amount and quality of purified RNA were monitored at various points throughout the purification process.

Three hundred nanograms of RiboMinus RNA fraction (Total RNA without eukaryotic rRNA) were then amplified and labeled using the Quick Amp Labeling kit of Agilent. The method consists of converting mRNA primed with a random primer containing a T7 promoter into double strand cDNA with MMLV-RT and then amplifying samples using a T7 RNA polymerase, which generates Cy3-labeled complementary (anti-sense) RNA (cRNA).

#### d. Microarrays hybridization

Cy3-labeled cRNAs and cDNAs obtained with both methods were used for hybridizations with Agilent Gene Expression Hybridization Kit (Agilent Technologies). Arrays were incubated at 65°C for 20 h in the hybridization chamber. After hybridization, arrays were washed according to the Agilent protocol. Genomic DNAs of *ER *Welgevonden and Gardel strains were labeled using BioPrime array CGH (Invitrogen) and then hybridized as positive control. cDNA samples (SCOTS 0×, 1×, 2×, 3×) from Gardel strain passage 39 and samples generated by IVT from Gardel strain passage 47 at 24 hpi and 96 hpi were hybridized on *ER *microarrays.

#### e. Microarrays analysis

Arrays were scanned and images were saved in TIFF format. The signal intensities of all spots on each image were quantified by Genepix pro 6.0 software (Molecular Devices Corporation, Downingtown, PA), and data were saved as ".txt" files for further analysis.

Data were log-transformed, mean-centered and reduced for an equal standard deviation between each slide using the GeneANOVA software [33]. The median value for each gene was calculated and correlation coefficients (R^2^) between the several conditions were calculated. Genes were considered detected when their intensity of fluorescence was superior or equal to 3 fold the mean of background intensity.

### *ER *Southern blots

Southern blots were carried out as described previously using the hybridization conditions explained by [34]. cDNAs produced at different times of infection (before SCOTS and after selective captures with 1×, 2× and 3× rounds of SCOTS) with rDNA16S and 23+5S were amplified by PCR digoxigenin (DIG)-labeling mix (Roche diagnostics, Meylan, France) according to manufacturer's instructions. Nylon membranes containing *ER *gDNA digested by *Hind*III were pre-hybridized at 50°C for 2 h in hybridization buffer [SSPE 6× (1× SSPE is 0.18 M NaCl, 10 mM NaH_2_PO_4_, and 1 mM EDTA {pH 7.7})], 0.5% sodium dodecyl sulfate [SDS], 2% (w/v) blocking reagent (Roche diagnostics). Probes were added to the hybridization buffer and incubated with the membranes for 16 h at 50°C. Blots were then washed twice in buffer containing 2× SSPE, 0.1% SDS (w/v). Colorimetric detection was performed with anti-DIG antibody conjugated to alkaline phosphatase (Roche) and BCIP/NBT Buffered Substrate (Sigma Aldrich, France).

### Real-time quantitative RT-PCR targeting ribosomal 16S genes

In order to evaluate the contaminant of *ER *rRNA16S transcripts in cDNA samples produced before and after SCOTS, qRT-PCR targeting *ER *16S gene was performed on each sample of Gardel strain passage 39. Sybergreen fluorescent master mix reagent (Applied Biosystem) was used for qRT-PCR. Primers 16S-F1 and 16S-R1 used were described in table 1 and the size of the amplicon was 121 pb. qRT-PCR was performed systematically on cDNA before and after each round of SCOTS (1×, 2×, 3×), using the following program: initial denaturation of 10 min at 95°C, followed by 35 cycles of denaturation at 95°C for 30 s, hybridization step at 60°C for 30 s and extension at 72°C for 60 s. In order to quantify the number of copies per sample, a standard curve was established using gDNA of Gardel strain passage 27 serially diluted (from 2.9 × 10^5 ^copies to 2.9 × 10^1^). Each sample was analyzed in duplicate.

### *ER *gene detection by RT-PCR and qRT-PCR in cDNA samples after selective capture

The presence of 5 genes in cDNA samples and their enrichment by SCOTS method were evaluated using RT-PCR and qRT-PCR amplifying small fragments of target genes (~120-300 pb) (table 1) that are *recA, ffh, rpoD, map1 *and *pCS20 *[18]. *map1 *gene of *ER *encodes an outer membrane protein (*major antigenic protein*) [35,36]. *ER *specific gene *pCS20 *is highly conserved and used as target for molecular *ER *detection [24]*. rpoD *gene encoding a polymerase sigma factor [37], *ffh *gene encoding a signal recognition particle protein and *recA *gene (recombinase A) [38] are three housekeeping genes involved in *ER *metabolism. The same primers and PCR conditions were used for RT and qRT-PCR.

For *ER *gene detection by RT-PCR, cDNA samples were diluted in order to assess optimal cDNA concentrations and then to observe the gradual increase of cDNA detection after the different rounds of SCOTS (from 1× to 3×). The dilution of cDNA samples depended on the time of infection: 10^3 ^fold for 24 hpi, 10^4 ^fold for 72 hpi, 10^5 ^for 96 and 120 hpi. These dilutions were used for all the target genes except *pCS20*. For *pCS20*, samples were not diluted for 24 hpi, and diluted 10, 10^3 ^and 10^2 ^fold for 72, 96 and 120 hpi. RT-PCR was performed systematically on cDNA before and after each round of SCOTS (1×, 2×, 3×) using the following program: initial denaturation of 3 min at 94°C followed by 40 cycles of denaturation at 94°C for 50 s, hybridization step at 60°C for 50 s and extension at 72°C for 50 s and a final extension of 7 min at 72°C. DNA of Gardel strain passage 18 was used as a positive control. PCR products were visualized by ethidium-bromide-stained revelation in agarose gels. qRT-PCRs targeting *map1, ffh and recA*, were performed on the ABI Prism 7000 (Applied Biosystems) in a total reaction volume of 25 μl. This reaction contained 2 μl of undiluted cDNA template (from the initial cDNA reverse transcribed (0.5 μg/μl) before capture and after SCOTS 3×). In order to quantify the number of copies per sample, a standard curve was made with the gDNA of *ER *(from 2.9 × 10^5 ^to 2.9 × 10^1 ^copies) as a template. Each sample was done in duplicate. A dissociation curve was produced in order to verify the presence of a single amplicon.

## Results

### Enrichment of bacterial cDNA after SCOTS

**Figure 1:**
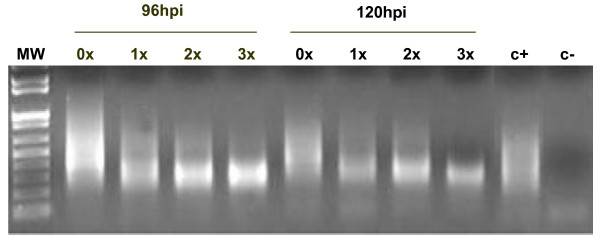
**NKpnI PCR amplicons of cDNAs of Gardel strain passage 39 after SCOTS**. 0×, 1×, 2× and 3×: cDNA amplicons of NKpnI PCR after 0, 1, 2 or 3 rounds of capture. C+: positive control cDNA with NKpnI tag. C-: Negative control (Water). MW: Molecular weight 100 pb DNA ladder. hpi: hours post infection

*ER *cDNAs were produced after 1×, 2×, and 3× SCOTS captures for each time point of infection. A PCR using *Kpn*I primer was performed on cDNAs after each capture. Figure 1 shows amplicons obtained after *Kpn*I PCR on Gardel strain passage 39, at 96 hpi and 120 hpi. The signal detected after capture confirmed the efficiency of the selective capture by high affinity hybridization of cDNA to gDNA. Moreover, we showed a progressive diminution of the size of the amplified transcribed sequences following successive captures. Similar results were obtained for other post infection time points (data not shown). These results indicated that same capture phenomena were observed independently from the time of infection.

**Figure 2:**
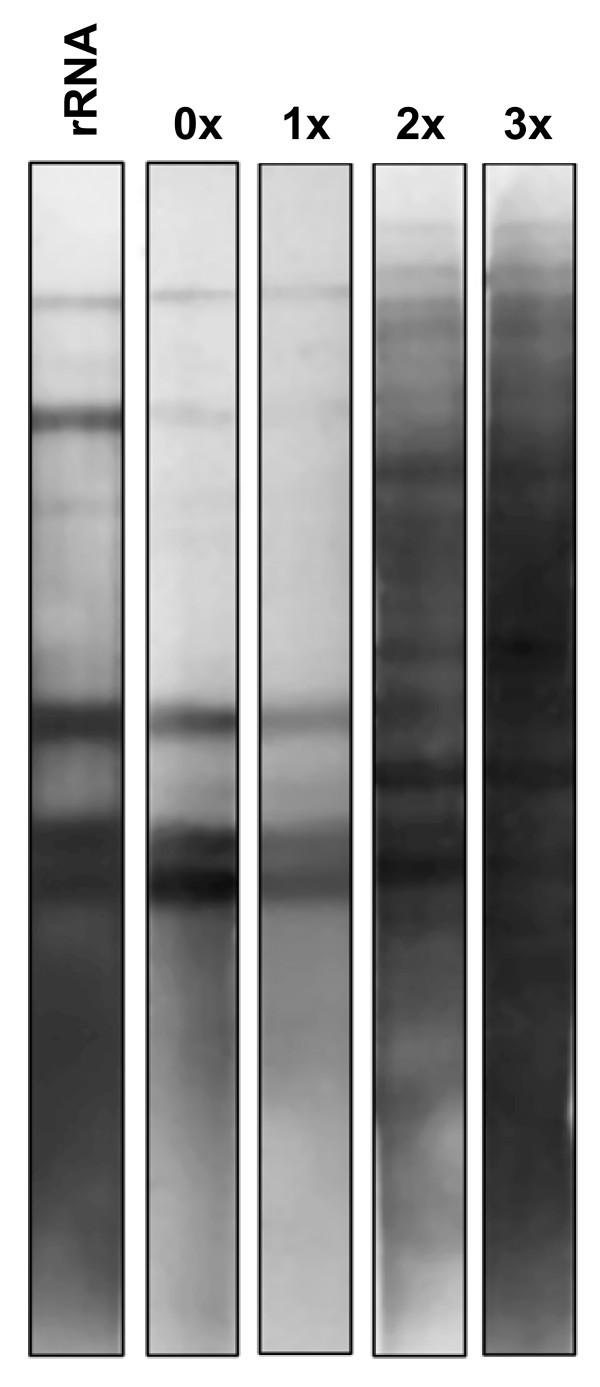
**Southern blot using DIG labeling cDNAs of Gardel strain at 96 hpi**. 0×, 1×, 2× and 3×: Southern blots showing cDNAs of Gardel strain passage 39 before and after one, two and three rounds of SCOTS. rRNA: Southern blot using DIG-rDNA 23S+5S and 16S.

Southern blots were done on cDNAs before and after each capture for each time point of infection. Southern blots obtained using cDNA at 96 hpi as probes on *Hind*III-digested *ER *gDNA are shown in figure 2. DIG-rDNA 23S+5S and 16S were used to reveal the bands corresponding to *ER *rDNA (lane 1). Five distinct bands corresponding to rDNA were observed both before capture and after the first capture (figure 2). After successive capture, the results showed a significant increase of the colorimetric signal that traduces a larger recognition of *ER *genes by DIG-cDNA at 96 hpi. These results demonstrated a progressive increase of bacterial cDNA complexity and amount following the successive rounds of captures. For other time points of infection, southern blots were done systematically and similar results were observed with a diminution of the ribosomal cDNA and an increase of the diversity of *ER *transcripts after the third capture (data not shown).

The diminution of the amplicon sizes after *Kpn*I PCR and results of Southern blot validates the enrichment of *ER *cDNA and diminution of ribosomal cDNA and eukaryotic contaminant.

### Quantification of ribosomal RNA 16S contaminant after SCOTS by qRT-PCR

**Figure 3:**
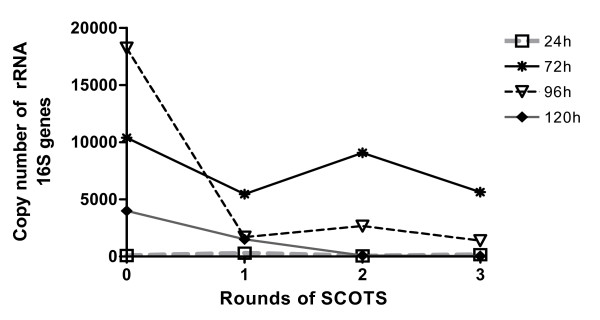
**Quantitative RT-PCR targeting *ER *rRNA 16S on cDNAs of Gardel strain after SCOTS at different time point post infection: 24, 72, 96 and 120 hours post infection**.

The quantification of cDNA corresponding to 16S cDNA contaminant was carried out before and after each round of SCOTS at the different time post infection by qRT-PCR (figure 3). Before capture, the amount of 16S cDNA contaminant varied depending on time points post infection and on the amount of bacteria. At 24 hpi, there was a few 16S cDNA contaminant (89 copies per sample). The range of contamination was between 4 × 10^3 ^to 18.2 × 10^3 ^copies for other time points post infection. We observed a decrease of 46%, 92% and 99% in ribosomal content after the third capture at 72, 96 and 120 hpi, with a final number of copies of 5.6 × 10^3^, 1.4 × 10^3 ^and 43 copies (figure 3). The main decrease was observed after the first capture. For 24 hpi, the initial number of 16S cDNA copies was already low (<90) and remained low after different captures.

### Detection of different *ER *transcribed sequences by RT-PCR and qRT-PCR before and after capture

Amplicons corresponding to RT-PCR targeting *map1, recA, rpoD, ffh and pCS20 *at different time points and following successive captures were shown in table 2. Before capture, the cDNAs of these genes were not detected. For *map1 *transcripts, one capture was sufficient to obtain a *map1 *amplicon at any time post infection. There was a significant and progressive increase of the amplification intensity after the second and third rounds, which demonstrated the enrichment of *map1 *transcripts (table 2). Depending on the gene and time point post infection considered, a positive signal was detected after the first (*i.e. ffh *at 24 hpi and *recA, ffh *at 72 hpi), second (*i.e. recA, ffh *at 96 hpi and *ffh *at 120 hpi) or third capture (*i.e. recA *at 24 hpi and 120 hpi). The increase of signal intensity was observed for all genes at any time point post infection after successive captures, except for *pCS20 *at 24 and 72 hpi. At 24 hpi, undiluted samples were used to detect *pCS20 *transcripts and there was no signal before capture. At 120 hpi, *pCS20 *amplification samples were diluted only at 10^-2 ^to observe enrichment, compared to the 10^-5 ^dilution used for the other genes. Globally, 3 rounds of capture were necessary to efficiently enrich the genes poorly expressed.

**Table 2:** Detection of *ER *specific genes by RT-PCR on total cDNA before and after SCOTS

Gene	24 hpi				72 hpi				96 hpi				120 hpi			
	0×	1×	2×	3×	0×	1×	2×	3×	0×	1×	2×	3×	0×	1×	2×	3×
*map*1	-	+	++	+++	-	+	++	+++	-	+	++	+++	-	+	++	+++
*recA*1	-	-	-	+	-	+	++	+++	-	-	++	+++	-	-	-	+++
*rpo*D	-	-	+	++	-	+	++	+++	-	-	++	+++	-	-	-	+++
*ffh*	-	+	++	+++	-	+	++	+++	-	-	++	+++	-	-	++	+++
*pCS20*	-	+	+	+	-	+	+	+	-	+	++	+++	-	+	++	+++

-: absence of amplicon after RT-PCR, +: Presence of amplicon after RT-PCR.

The number of (+) corresponded to the intensity of amplification signal. 0×, 1×, 2× and 3× corresponded to results obtained on cDNA before and after one, two and three rounds of capture respectively. hpi: hours post infection

**Figure 4:**
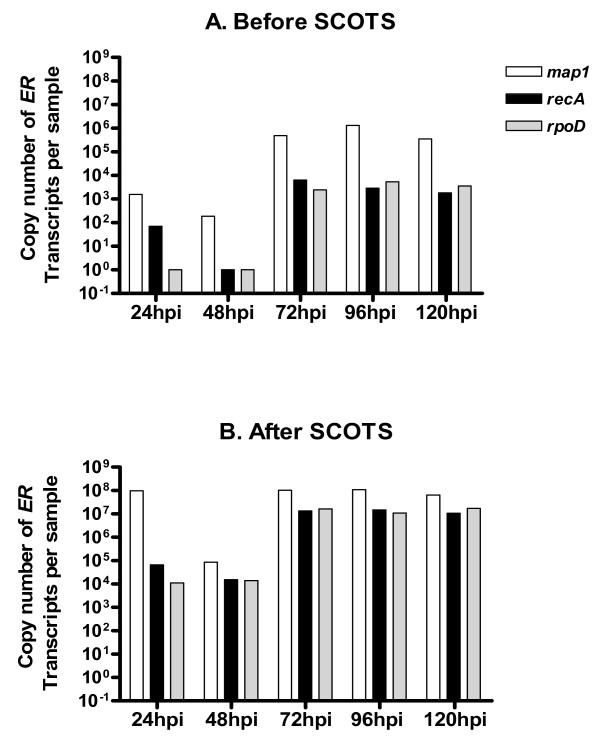
**Quantitative RT-PCR of *map1*, *recA *and *rpoD *genes on cDNA samples of Gardel strain before and after 3 rounds of capture. hpi: hours post infection**.

The qRT-PCR targeting *map1*, *recA *and *rpoD *transcripts before and after 3 rounds of capture allowed to quantify the enrichment due to SCOTS method. Results expressed as the number of transcripts for each gene obtained by qRT-PCR are presented in figure 4. Before capture, the number of transcripts was different depending on the target gene. For example, at 24 hpi there were 1.58 × 10^3 ^copies of *map1 *transcripts and only 70 and 10 copies of *recA *and *rpoD*. For all time points post infection, *map1 *was highly expressed compared to the 2 other genes (from 1 to 3 log10 higher than *recA *and *rpo*D) (figure 4).

After 3 captures at 24 hpi, there was enrichment around 1000 fold of the amount of *recA *transcripts (from 70 to 66 × 10^3^copies). For *map1 *transcripts, the number of copies increased from 1.58 × 10^3 ^to 1 × 10^8 ^copies after capture. Even when there was a single transcript per sample, for *rpoD *at 24 and 48 hpi and *recA *at 48 hpi, there was 11 × 10^3^, 14 × 10^3 ^copies and 15.4 × 10^3 ^copies after SCOTS. Before capture, there was an approximate 2 to 3 log10 difference of expression between *map1 *and *recA *or *rpoD*, whereas an approximate 1 log10 difference was measured after capture. The difference of expression between *map1 *and *recA *or *rpoD *was still observed after capture, whatever the culture time considered.

### Validation of selective captures using *ER *Microarrays

**Figure 5:**
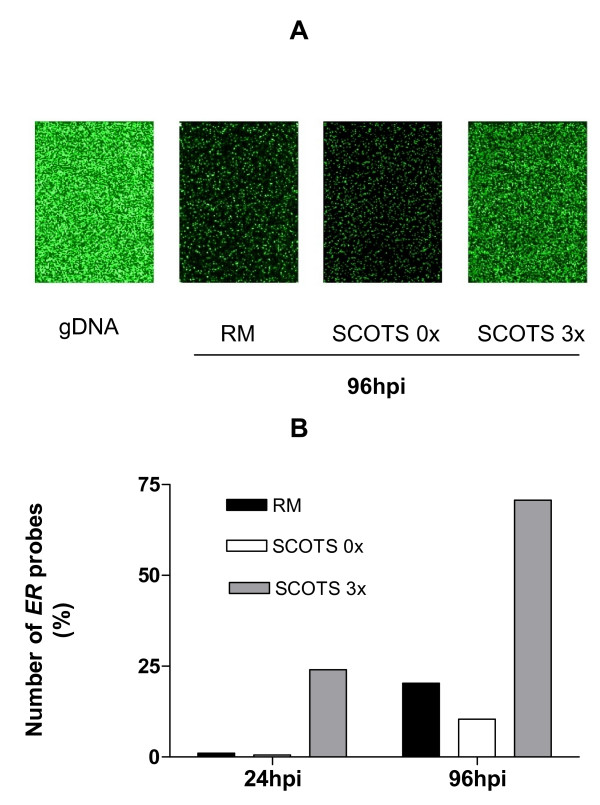
**Detection of probes using Gardel strain cDNAs generated after Ribominus (RM) and SCOTS treatment**. A: *ER *Microarray Hybridization with gDNA of Gardel strain passage 40, with cRNAs from Gardel strain passage 47 using RiboMinus purification (RM), with cDNAs from Gardel strain passage 39 before SCOTS (0×) and after 3 rounds of capture (3×). B: Percentage of *ER *detected probes at 24 and 96 hpi for cRNAs from Gardel strain passage 47 using RiboMinus purification (RM) with cDNAs from Gardel strain passage 39 before SCOTS (0×) and after 3 rounds of capture (3×).

To validate *ER *selective captures, whole genome microarrays of *ER *were used. Firstly, *ER *probes specificity was assessed by hybridizations with gDNA of Gardel and Welgevonden strains. Of the 1800 probes represented on our microarrays, 99.2 (1758/1772) and 99.1% (1757/1772) of probes were detected for Gardel and Welgevonden strains gDNA respectively (figure 5a). There was no detection of bovine probes corresponding to contaminants. When comparing gDNA hybridizations obtained for 5 replicates per probe on two microarrays slides, there was a high correlation coefficient (R^2 ^= 0.97).

Using *ER *microarrays, two methods for generating *ER *transcripts probes were evaluated: direct IVT from "RiboMinus" RNA fraction and cDNA classical random priming for SCOTS samples (figure 5). The percentages of genes detected using samples generated by these methods are presented in figure 5b. Before any treatment (SCOTS 0×), only a small fraction of transcripts could be detected (from 0.05 to 10.4%) for samples collected at 24 and 96 hpi. For early time post infection, there were only 19 genes (1%) detected by microarrays using the IVT method on "RiboMinus" RNA fraction, whereas 3 rounds of SCOTS allowed the detection of 24% of transcripts (figure 5b). Even at 96 hpi, the percentage of transcripts detected was low using the IVT method. SCOTS method allowed the detection of 7 and 3.5 fold more gene transcripts compared to untreated and RiboMinus samples. Thus, these results confirmed the efficiency of SCOTS method for transcriptomic analysis.

To evaluate a potential bias of the SCOTS method for differential gene expression analysis, we calculated the coefficients of correlation between successive captures on Gardel strain cDNAs at 96 hpi (table 3). The coefficients of correlation between each round of capture were 0.84, 0.98 and 0.98 respectively (table 3). The comparison of an untreated and a three SCOTS capture sample (R^2 ^= 0.7) indicated that SCOTS-mediated amplification was roughly linear.

**Table 3:** Correlation coefficient (R^2^) for gene detection by *ER *microarrays between the different rounds of SCOTS at 96 hpi

SCOTS	0×/1×	1×/2×	2×/3×	0×/3×
R^2^	0.84	0.98	0.98	0.7

## Discussion

Obligate intracellular pathogens are a challenge for functional genomic studies to identify genes involved in bacterial pathogenesis, especially at different stages of development. Until now, only a few studies have been performed on whole Rickettsiales transcriptomes, thus providing a good illustration of this constraint [39]. Global proteomic expression studies were preferentially used in order to enhance our knowledge on pathogenesis of obligate intracellular pathogens, such as *Ehrlichia, Rickettsia *and Anaplasma [40-44]. Proteomic studies also avoided the inconvenience of working with ribosomal prokaryotic and host cell contaminants.

In our Rickettsiales model, we postulated that pathogenicity determinants should be differentially expressed in the virulent strains of *ER *when compared to the same strain attenuated *in vitro *[45,46]. However, any future whole transcriptomic analysis of Rickettsiales bacteria will need to use efficient tools to eliminate both host cells contaminant and prokaryotic ribosomal transcripts.

A method of selection of prokaryotic transcripts, the RiboMinus method, based on the removal of eukaryotic ribosomal RNA, was tested for our model. No or few genes were detected on microarrays at any time post infection when using RiboMinus cRNA samples. The failure of the method could be due to the interference of important amount of eukaryotic transcripts. Another strategy, combining removal of eukaryotic contaminants with subsequent random amplification of prokaryotic cDNA, was used previously for *Rickettsia conorii *and gave convincing results for microarrays analysis [39,47]. However, SCOTS method for microarrays analysis uses small amounts of initial cDNA (3 μg of total RNA) compared to this selective method (50 μg for MicrobENRICH) [47]. Thus, using SCOTS method, there was no limitation to produce biological samples and several different conditions (strains and time-points post infection) could be studied.

The innovative method of SCOTS, which has been used previously for non obligatory intracellular parasites [6], was adapted to our *ER *model to obtain adequate samples for further whole-genome transcripts profile analysis.

The size diminution of *ER *cDNAs following successive captures illustrates a mechanism of generation of small cDNAs due to the nature of the Taq polymerase. Moreover, it shows the capture of smaller transcribed sequences (around 400 to 200 bp) after each successive round of SCOTS. This phenomenon was observed previously by Graham and Clark-Curtiss for *Mycobacterium tuberculosis *[4]. In their study, they showed that although there were potential biases in representing total mRNA of *Mycobacterium *due to SCOTS method (normalization and enrichment of cDNAs), the use of random priming to create cDNA provided a variety of different transcripts, thereby decreasing potential losses during amplification, normalization and enrichment. A progressive enrichment of *ER *cDNA between the first and the third capture was observed by Southern blotting as observed with *Mycobacterium tuberculosis *[4].

Interference due to ribosomal contaminants in transcriptomic analysis was shown previously in a study comparing subtractive hybridization and SCOTS methods for *Mycobacterium avium *[48]. For initial samples, few signals were observed corresponding to rRNAs. After subtractive hybridization and SCOTS, an increased quantity of messenger RNAs was observed. The deficiency of detection before mRNA selection seemed to be due to the high amount of rRNAs. In our model, we also obtained a strong decrease of the quantity of ribosomal contaminant as demonstrated by Southern blots and qRT-PCR targeting 16S gene. In an independent experiment, similar results were observed for attenuated Gardel strain by qRT-PCR (data not shown). Even if there was still detection of 16S transcripts in samples after capture, the level of contamination was negligible compared to total *ER *cDNAs and should not hinder transcriptomic analysis.

The detection of all the 5 tested genes including bacterial housekeeping genes *rpoD*, *ffh *and *recA *by RT-PCR or qRT-PCR suggests that SCOTS method in *ER *is efficient enough to enhance gene detection. Depending on the gene studied, variable numbers of captures were necessary to detect the specific transcript, illustrating the differential expression of genes in relation to life cycle. Three captures are required in order to detect poorly/lowest expressed genes and used for further transcriptomic analysis. Results of *recA *and *rpoD *qRT-PCR demonstrated that SCOTS method allowed the detection of transcripts accounted even when present as a single copy for early time-points post infection. Thus, our results demonstrated the efficiency of the SCOTS method for further expression analysis of an intracellular pathogen at early time-points post infection where the amount of eukaryotic contaminants was high. Beside this study, we report that *map1 *gene was strongly expressed as demonstrated both by RT-PCR and qRT-PCR. In parallel, we showed that *pCS20 *was the lowest expressed gene independently of the time of infection. For example, there was no detection of *pCS20 *transcripts after RT-PCR at early time post infection on undiluted samples.

As multiple PCRs were used for SCOTS method, one could suggest that all the transcripts would have the same level after selective capture. In this study, we demonstrated by both RT-PCR and qRT-PCR that the differential levels of expression were still observed after SCOTS. In a previous study, transcriptional analysis of *S. enterica *serovar Typhi within the macrophage revealed approximately 300 genes up-regulated at the defined point post infection compared to the supernatant [6].

In order to finalize the validation of SCOTS method for our model, we used *ER *microarrays. We first hybridized Gardel and Welgevonden strains gDNA and observed that more than 99% of probes were detected. These microarrays offered an exciting opportunity to do the genome-wide- analysis of *ER *gene expression.

As previously shown by RT-PCR and qRT-PCR on a limited number of genes, our microarrays results with samples generated by SCOTS confirmed the efficiency of this method for our model. Thus, SCOTS method seems to be ideal for whole genome expression profiling of *ER*. This method is crucial for the study of early time-points post infection: 24% of ORFs could be detected whereas less than 1% was detected on untreated cDNA samples. For late time-points post infection, up to 70.7% of ORFs were detected after 3 rounds. Considering all the time points of infection, 80% of the annotated ORFs were detected in our model (data not shown), which is similar to what was previously observed with *Salmonella *Typhi within the macrophage [6]. Through the use of SCOTS, comparison of gene expression between *ER *stages of development and between virulent and attenuated strains could be done on the overall CDS. This allows the targeting of genes involved in the invasion of host cells, in metabolism associated with bacterial growth (cell wall biogenesis, energy production, translation, traduction) and in pathogenesis sensu stricto.

Moreover, microarrays results demonstrated for overall *ER *genes that there was a good correlation between expression of genes comparing any round of SCOTS at 96 hpi. The lowest coefficient of correlation (R^2 ^= 0.7) was between SCOTS 0× and 3× samples. This was mainly due to the absence of detection of several genes before capture which could be detected after 3 rounds: the percentage of genes detected increased from 10.4 (SCOTS 0) to 70.7% (SCOTS 3). For genes already detected before capture, their level of expression was saturated after capture diminishing the correlation between SCOTS 0× and 3×. For attenuated Gardel, SCOTS 0× and 3× samples (96 hpi) were hybridized on *ER *microarrays and a higher correlation coefficient (R^2 ^= 0.87) was observed (data not shown). This preliminary result on attenuated Gardel strain, confirmed the limited bias due to SCOTS method on the gene expressions. Our microarrays data supported results obtained by qRT-PCR on *map1*, *recA *and *rpoD *(at any time-point post infection). The differential of gene expression diminished but was still detected before and after capture. However, for further transcriptomic analysis, we will focus on genes strongly differentially expressed or presence/absence of genes in order to target genes mainly involved in pathogenesis [39,49]. In our model, difference of gene expression will be assessed by hybridization of SCOTS cDNA on total *ER *microarrays and then validated by qRT-PCR on untreated cDNA.

## Conclusions

Our study reported herein demonstrated that SCOTS method has proven to be suitable for microarray-based transcriptome analysis of *ER *and as such can be potentially applicable to other obligate intracellular bacteria. SCOTS method avoids interferences due to host cells and prokaryotic ribosomal contaminants. Moreover, it allows the enhancement of specific transcripts and induced a limited bias in their relative amount. Thus, SCOTS method will offer the opportunity to study molecular mechanisms that take place in early stages of *ER *infection and to identify genes involved in the pathogenesis of this obligate intracellular bacterium.

## Authors' contributions

Conceived and designed the experiments: LE, FD, DFM, BM, RF, AV, PB, DM, TL, NV. Performed the experiments: LE, VP, CS, VM, NV. Analyzed the data: LE, DFM, BM, NV. Contributed reagents/materials/analysis tools: LE, FD, BM, AV. Wrote the paper: LE, FD, DFM, TL, NV. All authors read and approved the final manuscript.

## References

[B1] Belland RJ Zhong G Crane DD Hogan D Sturdevant D Sharma J Beatty WL Caldwell HD Genomic transcriptional profiling of the developmental cycle of Chlamydia trachomatis Proc Natl Acad Sci USA 2003 100 14 8478 8483 10.1073/pnas.1331135100 12815105 PMC166254

[B2] Hinton JC Hautefort I Eriksson S Thompson A Rhen M Benefits and pitfalls of using microarrays to monitor bacterial gene expression during infection Curr Opin Microbiol 2004 7 3 277 282 10.1016/j.mib.2004.04.009 15196496

[B3] Alland D Kramnik I Weisbrod TR Otsubo L Cerny R Miller LP Jacobs WR Jr Bloom BR Identification of differentially expressed mRNA in prokaryotic organisms by customized amplification libraries (DECAL): the effect of isoniazid on gene expression in Mycobacterium tuberculosis Proc Natl Acad Sci USA 1998 95 22 13227 13232 10.1073/pnas.95.22.13227 9789070 PMC23765

[B4] Graham JE Clark-Curtiss JE Identification of Mycobacterium tuberculosis RNAs synthesized in response to phagocytosis by human macrophages by selective capture of transcribed sequences (SCOTS) Proc Natl Acad Sci USA 1999 96 20 11554 11559 10.1073/pnas.96.20.11554 10500215 PMC18072

[B5] Daigle F Graham JE Curtiss R Identification of Salmonella typhi genes expressed within macrophages by selective capture of transcribed sequences (SCOTS) Mol Microbiol 2001 41 5 1211 1222 10.1046/j.1365-2958.2001.02593.x 11555299

[B6] Faucher SP Porwollik S Dozois CM McClelland M Daigle F Transcriptome of Salmonella enterica serovar Typhi within macrophages revealed through the selective capture of transcribed sequences Proc Natl Acad Sci USA 2006 103 6 1906 1911 10.1073/pnas.0509183103 16443683 PMC1413645

[B7] Baltes N Buettner FF Gerlach GF Selective capture of transcribed sequences (SCOTS) of Actinobacillus pleuropneumoniae in the chronic stage of disease reveals an HlyX-regulated autotransporter protein Vet Microbiol 2007 123 1-3 110 121 10.1016/j.vetmic.2007.03.026 17466471

[B8] Baltes N Gerlach GF Identification of genes transcribed by Actinobacillus pleuropneumoniae in necrotic porcine lung tissue by using selective capture of transcribed sequences Infect Immun 2004 72 11 6711 6716 10.1128/IAI.72.11.6711-6716.2004 15501809 PMC523062

[B9] Provost A Bezuidenhout JD The historical background and global importance of heartwater Onderstepoort J Vet Res 1987 54 3 165 169 3329308

[B10] Barré N Uilenberg G Morel PC Camus E Danger of introducing heartwater onto the American mainland: potential role of indigenous and exotic Amblyomma ticks Onderstepoort J Vet Res 1987 54 3 405 417 3329328

[B11] Uilenberg G Experimental transmission of Cowdria ruminantium by the Gulf coast tick Amblyomma maculatum: danger of introducing heartwater and benign African theileriasis onto the American mainland Am J Vet Res 1982 43 7 1279 1282 6808870

[B12] Jongejan F Wassink LA Lack of cross-protection between Cowdria ruminantium and Ehrlichia phagocytophila Rev Elev Med Vet Pays Trop 1991 44 4 425 428 1843823

[B13] Mahan SM Allsopp B Kocan KM Palmer GH Jongejan F Vaccine strategies for Cowdria ruminantium infections and their application to other ehrlichial infections Parasitol Today 1999 15 7 290 294 10.1016/S0169-4758(99)01468-4 10377533

[B14] Reddy GR Sulsona CR Harrison RH Mahan SM Burridge MJ Barbet AF Sequence heterogeneity of the major antigenic protein 1 genes from Cowdria ruminantium isolates from different geographical areas Clin Diagn Lab Immunol 1996 3 4 417 422 8807206 10.1128/cdli.3.4.417-422.1996 PMC170360

[B15] Zweygarth E Josemans AI Van Strijp MF Lopez-Rebollar L Van Kleef M Allsopp BA An attenuated Ehrlichia ruminantium (Welgevonden stock) vaccine protects small ruminants against virulent heartwater challenge Vaccine 2005 23 14 1695 1702 10.1016/j.vaccine.2004.09.030 15705474

[B16] Jongejan F Zandbergen TA Wiel PA van de de Groot M Uilenberg G The tick-borne rickettsia Cowdria ruminantium has a Chlamydia-like developmental cycle Onderstepoort J Vet Res 1991 58 4 227 237 1780122

[B17] Frutos R Viari A Ferraz C Bensaid A Morgat A Boyer F Coissac E Vachiery N Demaille J Martinez D Comparative genomics of three strains of Ehrlichia ruminantium: a review Ann N Y Acad Sci 2006 1081 417 433 10.1196/annals.1373.061 17135545

[B18] Frutos R Viari A Ferraz C Morgat A Eychenie S Kandassamy Y Chantal I Bensaid A Coissac E Vachiery N Demaille J Martinez D Comparative genomic analysis of three strains of Ehrlichia ruminantium reveals an active process of genome size plasticity J Bacteriol 2006 188 7 2533 2542 10.1128/JB.188.7.2533-2542.2006 16547041 PMC1428390

[B19] Frutos R Viari A Vachiery N Boyer F Martinez D Ehrlichia ruminantium: genomic and evolutionary features Trends Parasitol 2007 23 9 414 419 10.1016/j.pt.2007.07.007 17652027

[B20] Uilenberg G Camus E Barre N [A strain of Cowdria ruminantium isolated in Guadeloupe (French West Indies)] Rev Elev Med Vet Pays Trop 1985 38 1 34 42 3837922

[B21] Bezuidenhout JD Paterson CL Barnard BJ In vitro cultivation of Cowdria ruminantium Onderstepoort J Vet Res 1985 52 2 113 120 3900854

[B22] Martinez D Swinkels J Camus E Jongejan F [Comparison between 3 antigens for the serodiagnosis of heartwater disease by indirect immunofluorescence] Rev Elev Med Vet Pays Trop 1990 43 2 159 166 2092349

[B23] Vachiery N Lefrancois T Esteves I Molia S Sheikboudou C Kandassamy Y Martinez D Optimisation of the inactivated vaccine dose against heartwater and in vitro quantification of Ehrlichia ruminantium challenge material Vaccine 2006 24 22 4747 4756 10.1016/j.vaccine.2006.03.031 16621174

[B24] Martinez D Vachiery N Stachurski F Kandassamy Y Raliniaina M Aprelon R Gueye A Nested PCR for detection and genotyping of Ehrlichia ruminantium: use in genetic diversity analysis Ann N Y Acad Sci 2004 1026 106 113 10.1196/annals.1307.014 15604477

[B25] Martinez D Maillard JC Coisne S Sheikboudou C Bensaid A Protection of goats against heartwater acquired by immunisation with inactivated elementary bodies of Cowdria ruminantium Vet Immunol Immunopathol 1994 41 1-2 153 163 10.1016/0165-2427(94)90064-7 8066991

[B26] Perez JM Martinez D Debus A Sheikboudou C Bensaid A Development of an in vitro cloning method for Cowdria ruminantium Clin Diagn Lab Immunol 1997 4 5 620 623 9302217 10.1128/cdli.4.5.620-623.1997 PMC170611

[B27] Froussard P A random-PCR method (rPCR) to construct whole cDNA library from low amounts of RNA Nucleic Acids Res 1992 20 11 2900 10.1093/nar/20.11.2900 1614887 PMC336952

[B28] Daigle F Hou JY Clark-Curtiss JE Microbial gene expression elucidated by selective capture of transcribed sequences (SCOTS) Methods Enzymol 2002 358 108 122 full_text 12474381 10.1016/s0076-6879(02)58083-6

[B29] Hahn WE Pettijohn DE Van Ness J One strand equivalent of the Escherichia coli genome is transcribed: complexity and abundance classes of mRNA Science 1977 197 4303 582 585 10.1126/science.327551 327551

[B30] Ko MS Ko SB Takahashi N Nishiguchi K Abe K Unbiased amplification of a highly complex mixture of DNA fragments by 'lone linker'-tagged PCR Nucleic Acids Res 1990 18 14 4293 4294 10.1093/nar/18.14.4293 2377489 PMC331231

[B31] Rouillard JM Zuker M Gulari E OligoArray 2.0: design of oligonucleotide probes for DNA microarrays using a thermodynamic approach Nucleic Acids Res 2003 31 12 3057 3062 10.1093/nar/gkg426 12799432 PMC162330

[B32] Hubbard TJ Aken BL Ayling S Ballester B Beal K Bragin E Brent S Chen Y Clapham P Clarke L Coates G Fairley S Fitzgerald S Fernandez-Banet J Gordon L Graf S Haider S Hammond M Holland R Howe K Jenkinson A Johnson N Kahari A Keefe D Keenan S Kinsella R Kokocinski F Kulesha E Lawson D Longden I Megy K Meidl P Overduin B Parker A Pritchard B Rios D Schuster M Slater G Smedley D Spooner W Spudich G Trevanion S Vilella A Vogel J White S Wilder S Zadissa A Birney E Cunningham F Curwen V Durbin R Fernandez-Suarez XM Herrero J Kasprzyk A Proctor G Smith J Searle S Flicek P Ensembl 2009 Nucleic Acids Res 2009 37 Database D690 697 10.1093/nar/gkn828 19033362 PMC2686571

[B33] Didier G Brezellec P Remy E Henaut A GeneANOVA--gene expression analysis of variance Bioinformatics 2002 18 3 490 491 10.1093/bioinformatics/18.3.490 11934752

[B34] Bekker CP Postigo M Taoufik A Bell-Sakyi L Ferraz C Martinez D Jongejan F Transcription analysis of the major antigenic protein 1 multigene family of three in vitro-cultured Ehrlichia ruminantium isolates J Bacteriol 2005 187 14 4782 4791 10.1128/JB.187.14.4782-4791.2005 15995193 PMC1169525

[B35] Allsopp MT Dorfling CM Maillard JC Bensaid A Haydon DT van Heerden H Allsopp BA Ehrlichia ruminantium major antigenic protein gene (map1) variants are not geographically constrained and show no evidence of having evolved under positive selection pressure J Clin Microbiol 2001 39 11 4200 4203 10.1128/JCM.39.11.4200-4203.2001 11682561 PMC88518

[B36] van Vliet AH Jongejan F van Kleef M Zeijst BA van der Molecular cloning, sequence analysis, and expression of the gene encoding the immunodominant 32-kilodalton protein of Cowdria ruminantium Infect Immun 1994 62 4 1451 1456 8132352 10.1128/iai.62.4.1451-1456.1994 PMC186301

[B37] Savli H Karadenizli A Kolayli F Gundes S Ozbek U Vahaboglu H Expression stability of six housekeeping genes: A proposal for resistance gene quantification studies of Pseudomonas aeruginosa by real-time quantitative RT-PCR J Med Microbiol 2003 52 Pt 5 403 408 10.1099/jmm.0.05132-0 12721316

[B38] Takle GW Toth IK Brurberg MB Evaluation of reference genes for real-time RT-PCR expression studies in the plant pathogen Pectobacterium atrosepticum BMC Plant Biol 2007 7 50 10.1186/1471-2229-7-50 17888160 PMC2151947

[B39] Renesto P Rovery C Schrenzel J Leroy Q Huyghe A Li W Lepidi H Francois P Raoult D Rickettsia conorii transcriptional response within inoculation eschar PLoS ONE 2008 3 11 e3681 10.1371/journal.pone.0003681 18997861 PMC2577010

[B40] Ge Y Rikihisa Y Identification of novel surface proteins of Anaplasma phagocytophilum by affinity purification and proteomics J Bacteriol 2007 189 21 7819 7828 10.1128/JB.00866-07 17766422 PMC2168727

[B41] Wang X Kikuchi T Rikihisa Y Proteomic identification of a novel Anaplasma phagocytophilum DNA binding protein that regulates a putative transcription factor J Bacteriol 2007 189 13 4880 4886 10.1128/JB.00318-07 17483233 PMC1913470

[B42] Ogawa M Renesto P Azza S Moinier D Fourquet P Gorvel JP Raoult D Proteome analysis of Rickettsia felis highlights the expression profile of intracellular bacteria Proteomics 2007 7 8 1232 1248 10.1002/pmic.200600721 17385819

[B43] Renesto P Azza S Dolla A Fourquet P Vestris G Gorvel JP Raoult D Proteome analysis of Rickettsia conorii by two-dimensional gel electrophoresis coupled with mass spectrometry FEMS Microbiol Lett 2005 245 2 231 238 10.1016/j.femsle.2005.03.004 15837377

[B44] Huang H Lin M Wang X Kikuchi T Mottaz H Norbeck A Rikihisa Y Proteomic analysis of and immune responses to Ehrlichia chaffeensis lipoproteins Infect Immun 2008 76 8 3405 3414 10.1128/IAI.00056-08 18490460 PMC2493228

[B45] Jongejan F Protective immunity to heartwater (Cowdria ruminantium infection) is acquired after vaccination with in vitro-attenuated rickettsiae Infect Immun 1991 59 2 729 731 1987089 10.1128/iai.59.2.729-731.1991 PMC257822

[B46] Martinez D Analysis of the immune response of ruminants to Cowdria ruminantium infection 1997 Utrecht: Utrecht University

[B47] La MV Francois P Rovery C Robineau S Barbry P Schrenzel J Raoult D Renesto P Development of a method for recovering rickettsial RNA from infected cells to analyze gene expression profiling of obligate intracellular bacteria J Microbiol Methods 2007 71 3 292 297 10.1016/j.mimet.2007.09.017 17964675

[B48] Hou JY Graham JE Clark-Curtiss JE Mycobacterium avium genes expressed during growth in human macrophages detected by selective capture of transcribed sequences (SCOTS) Infect Immun 2002 70 7 3714 3726 10.1128/IAI.70.7.3714-3726.2002 12065514 PMC128060

[B49] Faucher SP Curtiss R Daigle F Selective capture of Salmonella enterica serovar typhi genes expressed in macrophages that are absent from the Salmonella enterica serovar Typhimurium genome Infect Immun 2005 73 8 5217 5221 10.1128/IAI.73.8.5217-5221.2005 16041043 PMC1201185

